# Correlation between Central Memory T Cell Expression and Proinflammatory Cytokine Production with Clinical Presentation of Multibacillary Leprosy Relapse

**DOI:** 10.1371/journal.pone.0127416

**Published:** 2015-05-19

**Authors:** Danuza Esquenazi, Iris Maria Peixoto Alvim, Roberta Olmo Pinheiro, Eliane Barbosa de Oliveira, Lilian de Oliveira Moreira, Euzenir Nunes Sarno, Jose Augusto da Costa Nery

**Affiliations:** 1 Leprosy Laboratory, Oswaldo Cruz Institute – FIOCRUZ, Rio de Janeiro, Brazil; 2 Laboratory of Cellular Microbiology, Oswaldo Cruz Institute – FIOCRUZ, Rio de Janeiro, Brazil; 3 Department of Pathology and Laboratories, School of Medical Sciences, State University of Rio de Janeiro - UERJ, Rio de Janeiro, Brazil; The University of Melbourne, AUSTRALIA

## Abstract

**Background:**

Despite the efficacy of multidrug therapy, surviving *Mycobacterium leprae* causes relapse in some leprosy patients, and these patients present signs and symptoms of disease after healing. This study focused on the cellular immune response in relapsed multibacillary patients but also included non-relapsed multibacillary cured individuals, newly diagnosed and untreated multibacillary patients, paucibacillary patients just before the beginning of treatment, and voluntary healthy individuals for comparative analysis.

**Methodology/Principal Findings:**

Inhibition of CD86 expression in the blood-derived monocytes and dendritic cells of relapsed multibacillary patients, either *ex vivo* or after *M*. *leprae* antigen stimulation was observed by flow cytometry. In addition, no significant changes in Interferon-gamma (IFN-γ) expression were observed in 5-day culture supernatants of relapsed patients in response to *M*. *leprae*, neither before nor after treatment, as measured by ELISA. However, these patients demonstrated a significant increase in central memory CD4+ and CD8+ *M*. *leprae*-specific T cells, as assessed by multiparametric flow cytometry. The increase in frequency of central memory T cells in relapsed patients strongly correlated with the bacillary index and the number of skin lesions observed in these subjects. Moreover, cytokine multiplex analysis demonstrated significant antigen-specific production of Interlukin-1beta (IL-1b), IL-6, and Tumour Necrosis Factor (TNF) in the relapsed group with extremely low IL-10 production, which resulted in a high TNF/IL-10 ratio.

**Conclusions/Significance:**

Inhibition of CD86 expression may function to reduce effector T cell responses against the *M*. *leprae* antigen. Furthermore, the predominance of central memory T cells in association with the high TNF/IL-10 ratio and no observed IFN-γ production may be related to the pathogenesis of relapse in multibacillary leprosy. Therefore, our findings may be a direct result of the clinical presentation, including a number of skin lesions and bacterial load, of relapsed patients. To our knowledge, this is the first study correlating immune response parameters with the clinical presentation of relapsed multibacillary patients.

## Introduction

Leprosy is an infectious disease caused by *Mycobacterium leprae* and approximately 200,000 new cases are still reported every year [1]. The disease initially affects the peripheral nerves and skin, with patients showing contrasting clinical, immunological, and pathological manifestations, despite minimal genetic variation among bacilli isolates [2]. Its clinical signs are related to both innate and adaptive immune responses, which either prevent invasion of bacterial components and infection or promote their development inside the host, thus giving rise to the pathogenesis of the disease. Protective cellular immunity inversely correlates with bacillary load, and the clinical spectrum ranges from strong *M*. *leprae*-specific cellular immunity in tuberculoid (TT) patients with localized disease, to humoral responses with a predominant lack of T cell immunity in lepromatous (LL) patients with disseminated skin lesions and borderline states (BT, BB and BL) positioned between polar forms. TT, BT, and indeterminate (I, initial phase of the disease) patients are grouped as paucibacillary (PB) forms, and LL, BL, and BB are grouped as multibacillary (MB) forms [3]. Although either a lack of response or an antigen-specific hyporesponsiveness appears to be a constant finding among MB patients, the recovery of specific proliferative responses has already been reported following long-term treatment and clinical cure [4,5]. However, while remaining anergic to the Mitsuda reaction in the lepromin skin test (LST), MB treated patients produce IL-2 and IFN-γ in response to *M*. *leprae* antigens, contrary to the cellular mediated anergy that exists in the majority of untreated MB patients [6].

Despite the partial recovery of cellular immunity after multidrug therapy (MDT), a number of cured individuals will relapse under the surveillance period, years after treatment and cure. Estimates of relapse rates vary widely within the regions affected by leprosy. The World Health Organization (WHO) estimates that the post-MDT relapse rates in endemic countries ranges from 3 to 4% of cases, with about 3400 relapses being reported in 2013 [1]. Relapse may be due to the growth of post-MDT surviving bacilli as a result of inappropriate or irregular therapy. The main differential diagnosis for relapse is reversal reactions (RR or type I reaction), drug resistance, and reinfection. In regards to MB forms, individuals with a higher bacillary index (BI>3) at pretreatment and negative LST are at higher risk for relapse. Moreover, accurate relapse diagnosis and identification of reactional states are crucial for preventing aggravation of neural damage as well as continued disease transmission in some situations [7,8].

It is possible that a gradual decline in the immunological mechanisms that contribute to recovery occurs in MB patients several years after treatment, thus favoring the growth of persistent dormant bacilli and subsequent leprosy relapse. Among such mechanisms, the activation of antigen presenting cells (APC), such as macrophages and dendritic cells (DC) by mycobacterial components may directly influence the quality of adaptive responses, by means of a discharge of mediators that determine the differentiation profile of the immune response. The adaptive immune response plays a critical role in infection control through generation of immunological memory, which composes the basis of protection against previously encountered antigens. Insight into the functionally different subsets of T cells has increased in recent years. Memory T cells encompass CD4 and CD8 T cells, which rapidly trigger effector functions and kill infected cells and secrete inflammatory cytokines. The expression of specific surface markers and effector functions, such as cytokine secretion and proliferation capacity distinguishes the heterogeneous population of memory T lymphocytes. Central memory T cells (T_CM_) preferentially reside in secondary lymphoid organs and mount recall responses to antigens. Although these cells lack immediate function, they rapidly proliferate and differentiate into effector T cells (T_EF_) following antigen stimulation. Effector memory T cells (T_EM_) are preferentially found in peripheral tissues, and provide immediate protection upon antigen challenge, by various mechanisms such as rapid production of effector cytokines [9].

A few studies have aimed at identifying the memory T cell subsets in leprosy. A previous report showed that in fresh and unstimulated blood leukocytes from leprosy patients, memory T cells predominated in the PB form of the disease and correlated with IFN-γ production. Among MB lepromatous patients, no preferential memory subset was observed [10]. However, the study did not use an experimental design that allowed discriminate identification of memory T cell subsets. Moreover, the phenotype, maintenance, T-cell memory functions (T_CM_ or T_EM_), T_EF_ phenotype, and the cytokines produced by these cells in leprosy relapse are not well known.

Therefore, the purpose of this work was to investigate parameters of both the innate and adaptive immune response in a group of MB relapsed patients, by comparing results obtained from newly diagnosed, untreated MB and PB patients, non-relapsed MB cured patients, and healthy donors.

## Material and Methods

### Ethics statement

The Institutional Ethics Committee of the Oswaldo Cruz Foundation/FIOCRUZ approved this study (permit protocol number 113/09) and an informed written consent was obtained from all individuals prior to specimen collection.

### Study Subjects

A total of 35 leprosy patients were enrolled in the study. This included 15 MB relapsed patients (8 LL, 5 BL, and 2 BB) who were re-starting MDT. All were residents of the Rio de Janeiro neighborhood, which is known to be endemic for leprosy. Non-relapsed cured MB individuals (n = 10; 5 LL, 3 BL, and 2 BB) under follow-up after treatment and showing no sign of relapse were also selected, as well as newly diagnosed and untreated MB (n = 10; 6 LL, 3 BL, and 1 BB) and PB patients (n = 10; all BT) diagnosed just before the beginning of MDT. All patients were diagnosed according to Ridley and Jopling criteria [11] and were attended at the Leprosy Outpatient Unit – FIOCRUZ, a reference center accredited by the Brazilian Ministry of Health, in Rio de Janeiro, Brazil. Routine procedures at our outpatient clinic include the following: dermatologic, neurologic and physical therapy assessment; histopathology of skin lesions; bacilloscopy (slit skin smear); and the lepromin skin test. We adopted a scoring system called “rating of lesions” based on the number of skin lesions presenting in our patients. The scores were assigned as follows: 0-5 lesions = 1, 6-10 lesions = 2, 11-20 lesions = 3 and > 20 lesions = 4. Voluntary healthy individuals (n = 10) living in the endemic leprosy area Rio de Janeiro were studied as the control group (HC). We adopted the following criteria for exclusion: pregnancy, recent vaccination, presence of infectious, autoimmune, and allergic diseases, diabetes, and hypertension.

### Blood isolation and culture

After venous puncture, blood was collected in heparinized tubes under endotoxin-free conditions, which was followed by Ficoll-Paque (GE Healthcare, Pittsburgh, PA, USA) density gradient centrifugation. Isolated peripheral blood mononuclear cells (PBMC) were washed in PBS and counted, and then cell purity was verified by the trypan blue (0.4%) exclusion assay (Sigma, St. Louis, MO, USA). PBMC were concentrated to 1×10^6^ cells/mL in AIM-V medium (Life Technologies, Grand Island, NY, USA) supplemented with 100 U/ml penicillin, 100 μg/ml streptomycin, and 2 mM glutamine (Life Technologies) and plated in 96-well U-bottom plates (Corning, Lowell, MA, USA) at 200 μL/well. The cells were then incubated at 37°C, 5% CO_2,_ and 90% relative humidity. Supernatants were harvested after 24 h, 72 h, and 5 days of culture and stored immediately at -70°C until use.

### Stimuli

Irradiated armadillo-derived *M*. *leprae* (20 μg/mL) (ML; provided from Colorado State University through the NIH/NIAID “Leprosy Research Support” grant under contract No. 1Al25469) were probe-sonicated immediately before use (>95% breakage) with a Vibra-Cell sonicator (Sonics, Newtown, CT, USA). The following reagents were used as positive control stimuli: 5 μg/mL phytohemagglutinin (PHA, Life Technologies), 1 μg/mL staphylococcal enterotoxin B (SEB, Sigma), and 1 ng/mL lipopolysaccharide (LPS, Sigma). Short-term cultures were performed in the presence of the costimulatory antibodies anti-CD28 and anti-CD49d (BD Biosciences, San Jose, CA, USA).

### IFN-γ ELISA

PBMC supernatants that were harvested after 5 days of culture were assayed for IFN-γ production by ELISA according to the manufacturer’s protocol (R&D Systems Inc., Minneapolis, MN, USA). Cytokine levels have been expressed as pg/mL of protein, and IFN-γ values in the experimental wells were subtracted from the values obtained in the unstimulated cultures (UNS). The cut-off value was 50 pg/mL and the detection limit of the assay was 4 pg/mL.

### Multiparametric Flow Cytometry

After 24, 48, 72, and 120 h-cultures, cells were washed twice in sorter buffer (PBS containing 1% FCS [Gibco BRL, USA] and 0.1% sodium azide [Sigma]) and blocked for 10 min with PBS containing FC-receptor bloking solution (BioLegend Inc., San Diego, CA, USA), then stained for 30 min in the dark with fluorochrome-conjugated monoclonal antibodies (mAbs) against the relevant cell surface antigens to characterize main leukocyte populations (anti-CD3 FITC, PE, and PerCP; anti-CD4 PerCP and APC; anti-CD8 PerCP; anti-CD14 FITC; anti-CD83 APC; anti-CD80 PerCP; and anti-CD86 PE) and cell activation status (anti-CD69 PE and Cy and anti-TLR2 APC) and to identify T cell phenotypes as naïve, memory, or effector (anti-CD45RO APC and anti-CD62L FITC). All mAbs were obtained from Becton Dickinson (BD Biosciences). After incubation at 4°C, cells were washed twice for 10 min, first with sorter buffer and then with PBS containing 0.1% sodium azide. Cells were then fixed with 1% paraformaldehyde (Sigma) and stored at 4°C in the dark. Cells were collected within 48 h on the FACSAria flow cytometer (BD Biosciences). Isotype controls were used to define the negative cell region, and 50,000 events were acquired for each sample. The data were analyzed using FlowJo version 8.2 software (Treestar, Ashland, OR, USA).

### Multiplex Determination of Cytokines

According to the manufacturer’s guidelines, inflammatory and immunomodulatory cytokines (IL-1β, IL-4, IL-5, IL-6, IL-8, IL-10, IL-13, IFN-γ, and TNF; R&D Systems, Minneapolis, MN, USA) were measured in unstimulated (UNS), ML-stimulated, SEB, and LPS- stimulated supernatants using a multiplex analyzer. IL-1β, IL-6, IL-8, and TNF were measured after 24 h culture, 3 days for IL-10, while IL-4, IL-5, IL-10, and IFN-γ were measured in 5-day culture supernatants. The procedure was performed as follows: after pre-wetting the filter with assay solution, the beads were washed twice with washing solution using 96-well multiscreen filter plates, a vacuum manifold, and a vacuum pump (Millipore, Billerica, MA, USA). Supernatant samples were added to the plates and incubated for 45 min at room temperature in the dark on a plate shaker. After three washes, detection antibody cocktail was added, and plates were incubated at room temperature in the dark for another 30 min on a plate shaker. After three washes, streptavidin-PE solution was added to each well and incubated for 10 min. After three washes, assay buffer was added and the plates were placed in the Bio-Plex system (MAPx-Luminex 100, Bio-Rad, Hercules, CA, USA) and analyzed by Bio-Plex Manager software (Bio-Rad). From each well, a minimum of 100 analyte-specific beads was analyzed for fluorescence. A curve fit was applied to each standard curve according to the manufacturer’s manual. Sample concentrations were interpolated from these standard curves for cytokine quantification. The values deemed as positive were as follows: 30 pg/mL for IL-8 and TNF; 32.5 pg/mL for IL-1β, IL-6, and IL-10; and 50 pg/mL for IL-4, IL-5, IFN-γ, and IL-13.

### Statistical and graphic analyses

The data were analyzed using GraphPad Prism version 5.0 (GraphPad, San Diego, CA, USA). Results are reported as median ± standard error of the median (SEM) or interquartile range (25-75% percentile). The nonparametric Kruskal-Wallis with post-test Dunns analysis was employed to determine differences between stimulated cells and unstimulated cells. The Mann–Whitney test was used to group comparisons, and the Pearson’s test was used for a correlation analysis. The statistical significance level was *p<* 0.05.

## Results

### Demographic and clinical data of the subjects included in the study

All 15 MB relapsed patients under follow-up in our study newly presented the signs and symptoms of the disease within an average 12.5-year timeframe (ranging between 2 and 22 years) upon discharge. No patient from this group presented any reaction episodes with indicative signs of RR, such as fever, malaise, anorexia or neuritis in both clinical and histopathology examination at relapse diagnosis. The mean age of these patients at relapse was 49.3 years old (ranging between 30 and 73 years), while the mean age of these same individuals was 36.9 years old (ranging between 13 and 55 years) at first leprosy disease (FLD). The average bacillary index (BI) at FLD was 3.8 (ranging between 2.75 and 4.75), and 3.9 (ranging between 2.25 and 5.57) at relapse. Approximately 47% (n = 7) of patients presented an increased BI between FLD and relapse. Clinically, a frequent finding in our patients was the higher number of lesions covering at least three areas of the body at relapse diagnosis when compared to the FLD. It is noteworthy that 60% of the relapsed patients (n = 9) presented a score of 4 based on our lesion scoring system, while in the untreated MB group the majority of patients (70%, n = 7) presented score of 3. The majority of the relapse patients (73.3%, n = 11) with a more than a 10-year interval to relapse were initially treated, specifically with a 24-dose MDT-MB therapy during the FLD. However, at relapse all patients were treated with a 12-dose MDT therapy, as currently recommended by the WHO (Table 1).

**Table 1:** Characteristics of multibacillary patients at the FLD and at relapse.

PATIENT ID			FIRST LEPROSY DISEASE (FLD)						RELAPSE					
*Cd*.	*Sex*	*Time of Rel*. *(ys*.*)*	*Age (ys*.*)*	*Diag*.	*BI*	*LST*	*Treatment regimen*	*Reaction*	*Age (ys*.*)*	*Diag*.	*BI*	*Rating of lesions*	*LST*	*Treatment regimen*
AA	M	10	47	LL	4.75	Neg	MDT-MB*	ENL	58	LL	3.75	4	Neg	MDT-MB
AB	M	19	13	LL	3.85	ND	MDT-MB*	-	32	LL	4.0	4	Neg	MDT-MB
AC	F	12	55	LL	3.55	Neg	MDT-MB*	ENL	67	LL	5.0	4	Neg	MDT-MB
AD	M	13	20	LL	4.0	Neg	MDT-MB*	-	33	LL	3.45	4	Neg	MDT-MB
AE	F	20	30	LL	ND	ND	MDT-MB*	-	49	LL	4.75	3	Neg	MDT-MB
AF	F	22	30	LL	3.85	ND	MDT-MB*	-	52	LL	5.57	4	Neg	MDT-MB
AG	F	16	44	LL	4.0	Neg	MDT-MB*	-	60	LL	5.5	3	Neg	MDT-MB
AH	M	11	23	LL	4.25	Neg	MDT-MB*	ENL	34	LL	3.25	2	Neg	MDT-MB
AI	M	2	51	LL	4.25	Neg	MDT-MB	ENL	53	BL	4.75	4	Neg	MDT-MB
AJ	F	17	52	LL	ND	ND	MDT-MB*	-	69	BL	3.57	3	Neg	MDT-MB
AK	M	14	34	BL	3.75	Neg	MDT-MB*	ENL	48	BL	3.25	3	Neg	MDT-MB
AL	M	3	28	BL	2.5	Neg	MDT-MB	ENL/RR	31	BL	3.0	2	Neg	MDT-MB
AM	M	20	53	LL	ND	Neg	MDT-MB*	-	73	BL	3.75	3	Neg	MDT-MB
AN	F	4	48	BB	2.5	Neg	MDT-MB	RR	52	BB	2.25	2	Neg	MDT-MB
AO	M	5	25	BB	2.75	Neg	MDT-MB	RR	30	BB	3.15	2	Neg	MDT-MB

Cd.: randomized code attributed for each patient in order to safeguard their identity; Rel.: relapse; M: male; F: female; Ys.: years; Diag.: histopathological diagnosis; LL: lepromatous leprosy; BL: borderline lepromatous leprosy; BB: borderline borderline leprosy; BI: bacteriological index; Rating of lesions: 0-5 lesions = 1; 6-10 lesions = 2; 11-20 lesions = 3 and >20 lesions = 4; LST: lepromin skin test; MDT-MB*: multidrug therapy - multibacillary leprosy (rifampicin 600 mg once a month, dapsone 100 mg daily, and clofazimine 300 mg once a month and 50 mg daily, duration = 24 doses); MDT-MB: multidrug therapy currently recognized by WHO (12 doses); ENL: erythema *nodosum leprosum*; RR: reversal reaction; Neg: negative; ND: not done.

The group of newly diagnosed and untreated patients consisted of 20 individuals (10 MB and 10 PB). These patients started MDT immediately at collection of peripheral blood for laboratory studies. Among the group of clinically cured individuals (n=10) at post-MDT yearly follow-up, the average healing period was 5.5 years (ranging between 1 and 10 years) and at disease diagnosis, all were MB. No signs of disease reactivation were observed in this study group, as determined by dermatologic examination (Table 2).

**Table 2:** Baseline patterns of newly diagnosed and untreated patients, cured leprosy patients, and healthy volunteers.

*Groups of individuals (n)*	*Diagnose (n)*	*Age (ys*.*) (range)*	*Sex (%)*	*BI (range)*	*Rating of lesions (n)*	*LST (n)*	*Healing period (ys*.*) (range)*
**Untreated (20)**							
MB (10)	LL (6), BL (3), BB (1)	37 (20-54)	M (70) F (30)	3.55 (2.75-4.25)	2 (1), 3 (6), 4 (3)	Neg (10)	-
PB (10)	BT (10)	44 (18-71)	M (60) F (40)	Neg	1 (10)	Neg (1) Pos (9)	-
**Cured (10)**	LL (5), BL (3), BB (2)	48 (26-76)	M (70) F (30)	-	-	Neg (10)	6.4 (1-10)
**Healthy controls (10)**	-	36 (23-54)	M (50) F (50)	-	-	-	-

(n): number of cases; Diagnosis, histopathological diagnosis; (ys.): years; BI: bacteriological index; Rating of lesions: 0-5 lesions = 1, 6-10 lesions = 2, 11-20 lesions = 3 and >20 lesions = 4; LST: lepromin skin test; M: male; F: female; Neg: negative; Pos: positive.

### Relapsed MB patients do not produce IFN-γ in response to *M*. *leprae*

**Fig 1:**
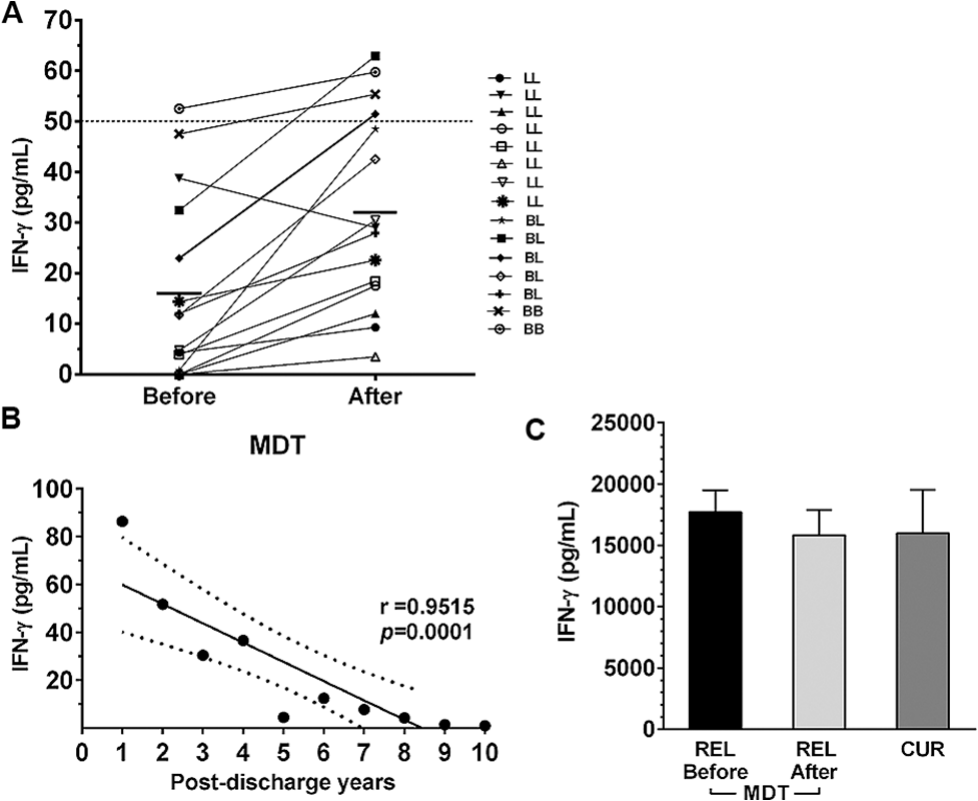
IFN-γ production in response to *M*. *leprae* in MB relapsed patients and cured individuals. IFN-γ levels after 5-day *ex vivo* PBMC stimulation with whole *M*. *leprae* (ML, 20 μg/mL) in relapsed multibacillary patients before and after MDT treatment. Horizontal lines show the median of the group (n=15). A). Correlation between levels of IFN-γ production and post-discharge years in clinically cured individuals under post-MDT follow-up. Each symbol represents one single individual. The numbers below the x-axis represent the number of years since cure. r = correlation coefficient; *p =* significance level (Pearson test, correlation analysis). B). IFN-γ production in response to 5 μg/mL PHA. The columns represent the median (range) of each tested relapsed group (REL and CUR, cured patients). C). ELISA; values ≥ 50 pg/mL are considered positive (Kruskal-Wallis test, comparison of stimulated cells with unstimulated cells; Mann–Whitney test, group comparisons).

First, to assess the ability of relapsed patients to produce IFN-γ *in vitro*, PBMC from these patients were cultured with ML and PHA, immediately upon confirmation of the relapse diagnosis and exclusion of reactional states, within 30 days from MDT completion upon discharge for healing. Among the 15 patients diagnosed as relapsed, only one who had been histologically classified as BB, was able to produce positive levels of IFN-γ (52.5 pg/mL) after *in vitro* ML stimulation. The majority of relapsed patients (93.3%, n=14) did not produce high levels of IFN-γ before (16.4 ± 4.7 pg/mL) or after treatment (32.7 ± 5.1 pg/mL; Fig 1A). Moreover, four (26.7%) patients showed IFN-γ production following completion of MDT. However, as shown in Fig 1A, although there was a significant difference (*p<* 0.05) in the median values obtained from relapsed patients at the studied situations, they were below the positive detection limit of the ELISA test (≥ 50 pg/mL). As expected, all PHA-stimulated cells (positive control) demonstrated high levels of IFN-γ production with median values of 15,9 ± 1,7 pg/mL (before) and 13,8 ± 2,0 pg/mL after treatment (Fig 1C).

### Clinically cured patients demonstrate reduced IFN-γ production that is proportional to healing time

To determine if there is a relationship between the ability of PBMC to produce IFN-γ and a given timeframe, the PBMC response of 10 MB leprosy patients to ML was examined within 1 to 10 years after completion of MDT. Only two individuals in this group demonstrated IFN-γ production in response to antigen stimulation within 1 and 2 years of discharge, showing levels of 86.4 and 51.7 pg/mL, respectively. All of the other patients with longer timeframes after completing MDT (8/10, 80%) did not produce IFN-γ levels above the cut-off of 50 pg/mL (r= 0.9515, *p=* 0.0001; Fig 1B). However, all patients produced high IFN-γ levels (14,9 ± 3,5) in response to the positive control mitogenic stimulus (PHA, Fig 1C).

### Inhibition of CD86 in monocytes and dendritic cells (DC) by *M*. *leprae* significantly increases in relapse

**Fig 2:**
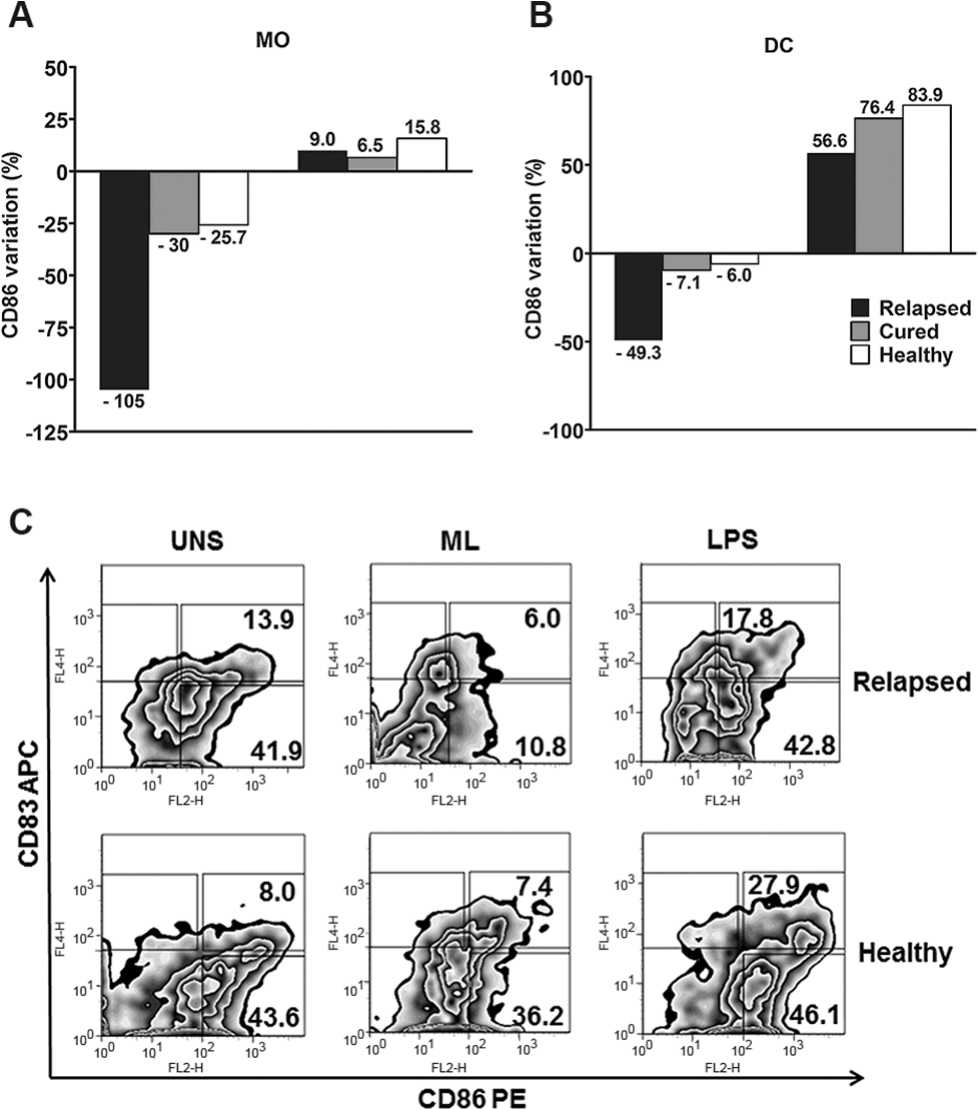
Significant inhibition of CD86 expression in monocytes and DC from MB relapsed leprosy patients. PBMC cultures stimulated with *M*. *leprae* (20 μg/mL) and LPS (1 μg/mL) for 24 h were analyzed by flow cytometry (FACSAria, BD). After gating the monocytes and DC region, based on forward and side scatter and the negative region defined with isotype control antibodies for each cell population, CD86 expression was obtained both in monocytes (CD3-/CD83-/CD14+; A) and DC (CD3-/CD14-/CD83+; B). On the left, the bars of both graphics correspond to antigen stimulation, while those on the right correspond to LPS stimulation, in accordance with the assessed groups. The results are expressed as percentage variation, calculated according to the following formula: [CD86 expression in ML-stimulated cultures (%) / CD86 expression in unstimulated cultures (%) × 100] −100; C). A representative example of the flow cytometric determination of CD86 expression in unstimulated (UNS), *M*. *leprae* stimulated (ML), and LPS stimulated monocytes, and DC from a relapsed patient (upper dot plots) and a healthy volunteer (lower dot plots). The numbers inside the dot plots represent the percentage of CD86 positive cells in monocytes (CD83-; lower right panels) and DC (CD83+; upper right panels).

In order to evaluate the initial phase of the immune response to ML in leprosy relapse, we analyzed the expression of molecules related to cellular activation (TLR2), antigen presentation (HLA-DR) and costimulation B7.1 (CD80) and B7.2 (CD86) in monocytes and DC from relapsed patients and other groups. PBMC were stained with specific mAbs in order to differentiate between monocytes and DC and also to verify the expression of surface receptors through multiparametric flow cytometry. B7.1 (CD80), HLA-DR and TLR2 expression was reduced in ML stimulated monocytes (CD3-/CD14+/CD83-) and DC (CD3-/CD83+/CD14-) from relapsed patients compared to healthy controls (*p<* 0.05). However, there was no significant difference in relation to clinically cured individuals (*p>* 0.05; data not shown). Both in ML-stimulated monocytes and DC, B7-2 (CD86) expression was reduced in all evaluated groups when compared to non-stimulated cultures. However, relapsed patients presented a higher percentage of CD86 inhibition both in monocytes and DC. These findings were strongly significant compared to the clinically cured patients and healthy controls (*p<* 0.001). When stimulated by LPS, the same cells, including those from relapsed patients, did not demonstrate ML triggered inhibition of CD86 expression (Fig 2A and 2B). A representative flow cytometric dot plot analysis of CD86 expression in monocytes (CD83-) and DC (CD83+) from a relapsed patient and a healthy volunteer confirmed the above findings (*p<* 0.001; Fig 2C).

### Predominance of central memory CD4+ and CD8+ T cells in leprosy relapse

**Fig 3:**
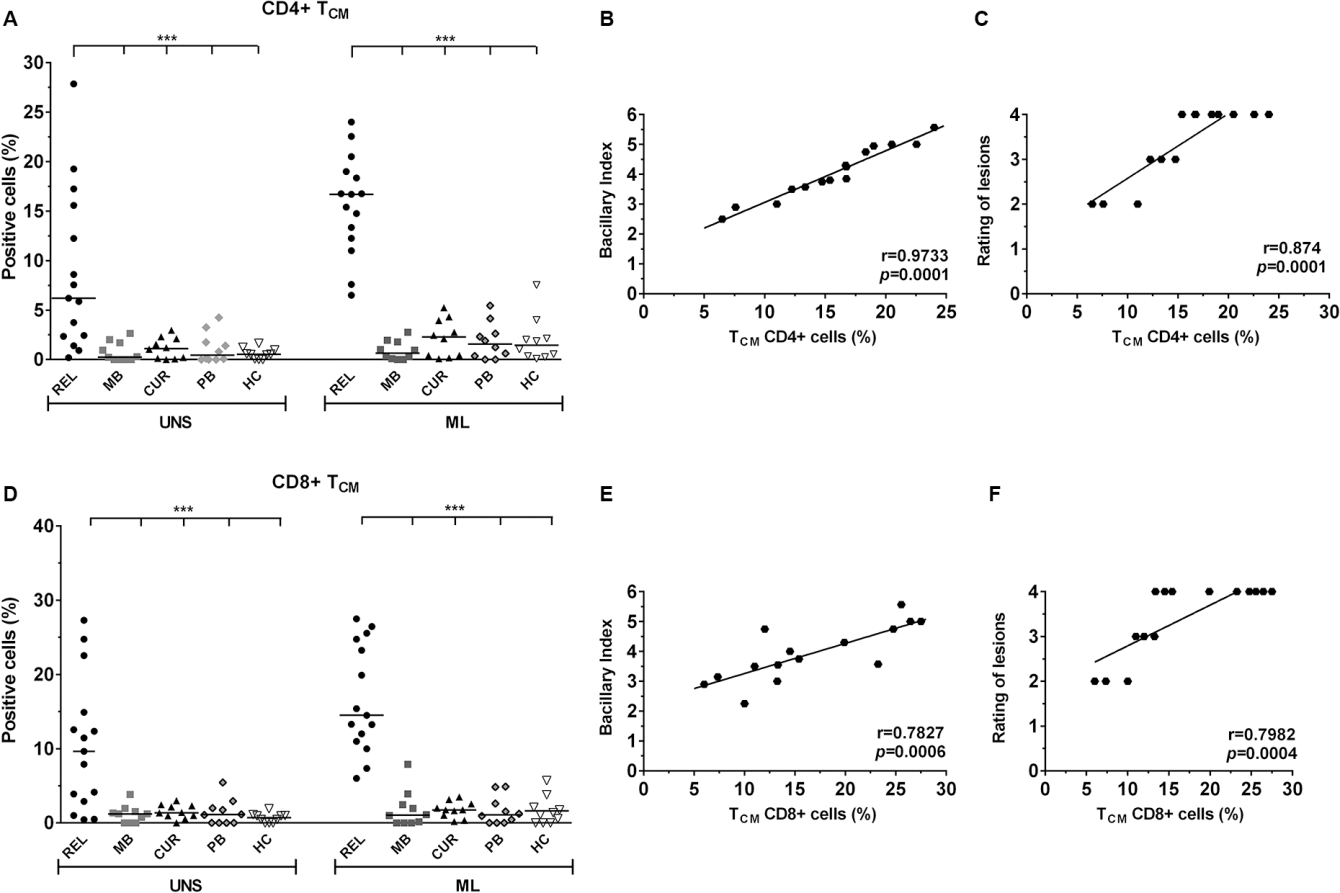
Increase in the frequency of CD4+ and CD8+ TCM lymphocytes in relapsed patients is related to BI and to the number of skin lesions. CD4+ and CD8+ TCM cells (CD69+/CD45RO+/CD62L+high; A and D) in response to in vitro 24-h PBMC stimulation with M. leprae (20 μg/mL) by multiparametric flow cytometry (FACSAria, BD). The results are expressed as percentage of positive cells, and the statistical differences are shown (***p< 0.001; UNS: unstimulated). The horizontal bars represent the median of each group. In each analysis, isotype controls were used to distinguish between positive and negative cells. SEB (1μg/mL) was positive in all tests (data not shown). The Kruskal-Wallis test was used for comparison of stimulated cells with unstimulated cells, and the Mann–Whitney test to group comparisons. Correlation analysis between CD4+ and CD8+ TCM and BI (B and E) and the number of lesions (C and F). Significant positivity is demonstrated (r = correlation coefficient; p= significance level; Pearson test).

To evaluate the phenotype of T cell subsets from relapsed patients, PMBC stimulated with ML or SEB and non-stimulated PMBC were stained with T_N_ (CD45RO-/CD62L+), T_EF_ (CD45RO-/CD62L-), T_CM_ (CD45RO+/CD62L+^high^) and T_EM_ (CD45RO+/CD62L-) lymphocyte specific mAbs. With the exception of T_N_ cells, the remaining subsets were analyzed for their ability to induce activation through the CD69 receptor, an early T cell activation marker. The frequencies obtained from each T cell subpopulation are shown in S1 Table. There were no observed differences in CD4+ and CD8+ T_N_ lymphocytes in relapsed and MB patients. Interestingly, the cured group showed a significant increase in the frequency of ML-specific CD8+ T_N_ cells (*p<* 0.05), but this was not observed in the CD4+ T cell subset. Furthermore, relapsed patients were unable to activate ML-specific clones of either of CD4+ and CD8+ T_EM_ or T_EF_ cells in any of these individuals. PB patients and HC subjects showed significant increases of CD4+ and CD8+ T_N_, T_EF_ and T_EM_ subpopulations in response to the antigen (*p<* 0.05). Remarkably, *ex vivo* CD4+ and CD8+ T_CM_ frequency in the relapsed group was increased (6.2 ± 2% and 9.6 ± 2.2% respectively) compared to all other groups. Likewise, when stimulated by ML, the frequency of the CD4+ (16.6 ± 1.2%) or at CD8+ (14.5 ± 1.9%) phenotype also increased significantly in comparison to the other groups (*p<* 0.001; S1 Table and Fig 3A and 3D).

### T_CM_ frequency correlates with the bacillary index (BI) and the number of lesions in relapsed patients

To determine whether the increased frequency of T_CM_ cells seen in relapsed patients correlated with clinical and laboratory findings, we evaluated a possible correlation of these subpopulations with BI and the number of skin lesions, within our lesion scoring system as described in Table 1. The correlation was strongly significant between CD4+ T_CM_ lymphocytes and BI (r= 0.9733, *p=* 0.0001), as well as the number of lesions (r= 0.874, *p=* 0.0001; Fig 3B and 3C). Although significant, CD8 + T_CM_ cells showed a lower correlation with both BI (r= 0.7827, *p=* 0.0006) and the number of lesions (r= 0.7982, p= 0.0004; Fig 3E and 3F), which may indicate that these subsets are responsible, at least in part, for the inflammatory state in relapsed patients. The positive control SEB was able to activate all T cell subsets (data not shown). To verify whether the formation of certain ML-specific T cell subpopulations depended on the period of antigen stimulation, the cells of 3 relapsed patients were further analyzed after 48, 72, and 120 h of culture. After the 20-48 h period, which was marked by the predominance of T_CM_, the extension of the culture period produced no impact on the development of ML-specific T_EF_ in these patients (data not shown).

### *M*. *leprae* induced significantly higher levels of IL-1β, IL-6 and TNF among relapsed patients

**Fig 4:**
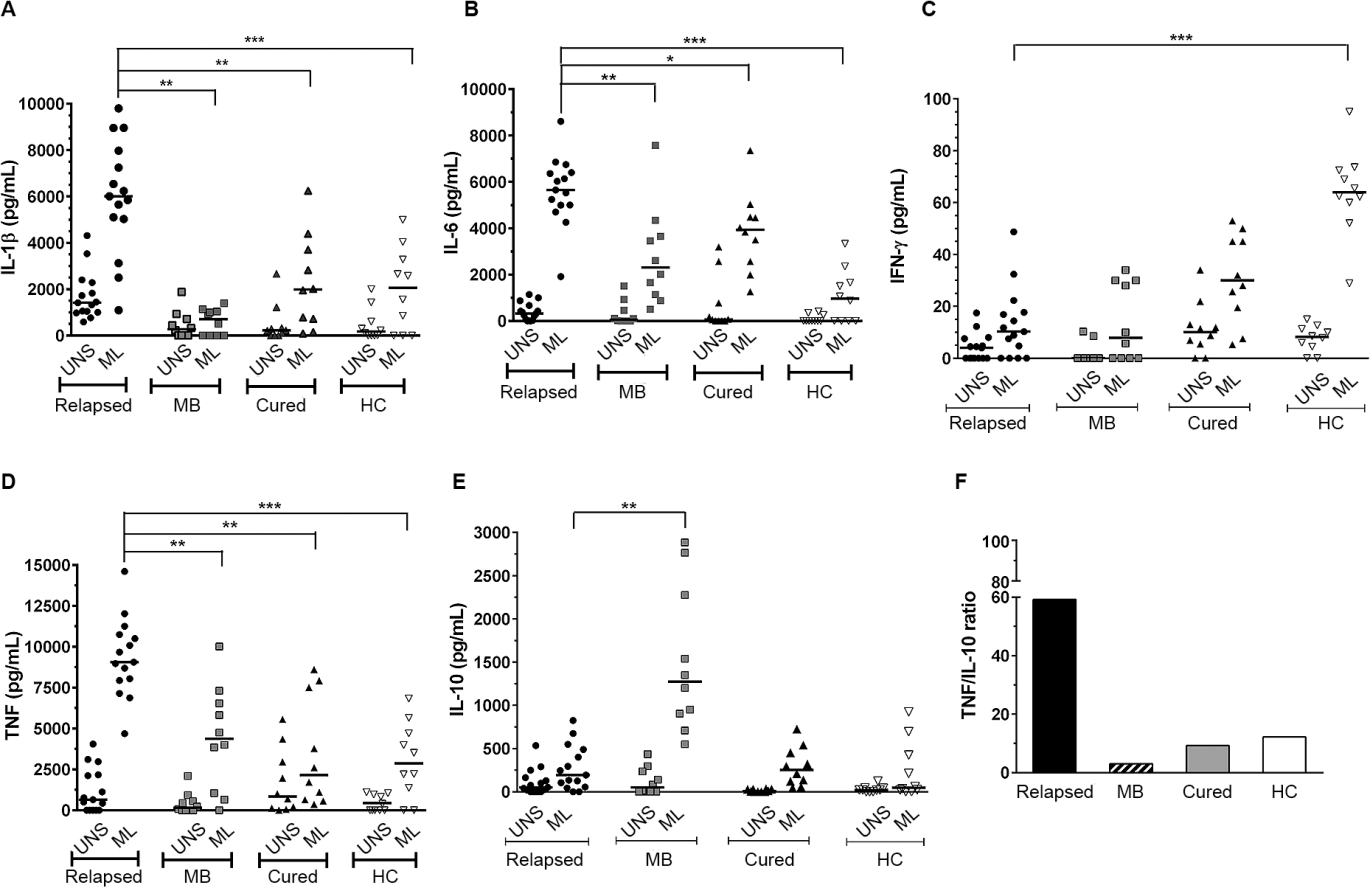
The cytokine profiles in response to *M*. *leprae*, TNF, and IL-10 are differentially induced in PBMC during relapse. Detection of IL-1β (A), IL-6 (B), IFN-γ (C), TNF (D) and IL-10 (E) in supernatant cultures was performed by multiplex assay. The results are expressed in pg/mL and the statistical differences are shown (*p<0.05, **p< 0.005 and ***p< 0.001; the Kruskal-Wallis test was used for comparison of stimulated cells with unstimulated cells and the Mann–Whitney test was used to group comparisons). UNS: unstimulated and ML: *M*. *leprae* stimulated cultures (20 μg/mL). MB: untreated multibacillary; HC: healthy controls. The horizontal bars represent the median of each group. SEB (1 μg/mL) was positive in all tests (data not shown). A high *M*. *leprae*-specific TNF/IL-10 ratio between relapsed patients and other groups, especially newly diagnosed and untreated MB patients was observed (F).

In order to evaluate the production of pro-inflammatory and immunomodulatory cytokines from relapsed patients, supernatants of ML-stimulated PBMC cultures were analyzed by multiplex assay for IL-1β, IL-4, IL-5, IL-6, IL-8, IFN-γ, TNF, IL-10, and IL-13 secretion. Relapsed patients produced significantly high levels of IL-1β (6,006 ± 634 pg/mL), IL-6 (5,650 ± 382 pg/mL), and TNF (9,064.5 ± 414 pg/mL) in response to antigen, when compared to newly diagnosed and untreated MB, cured, and HC groups (*p<* 0.05; Fig 4A, 4B and 4D). It is noteworthy that background production (unstimulated cultures; UNS) of IL-1β was also increased among these patients (1,418 ± 270 pg/mL), particularly in comparison to newly diagnosed and untreated MB patients (*p<* 0.05; Fig 4A and 4D). With the exception of healthy volunteers, IL-6 levels in ML-stimulated cultures were considerably high in all groups, especially in relapsed patients as previously mentioned (Fig 4B). Surprisingly, there was no significant IL-10 production in relapsed patients, however significant levels of this cytokine were verified only among newly diagnosed and untreated MB patients (1,375 ± 267 pg/mL; *p<* 0.001) under antigen stimulation (Fig 4E). Since TNF and IL-10 play opposite roles during mycobacterial infection, it was interesting to compare the production of these cytokines. Therefore, we evaluated ML-specific TNF/IL-10 ratio, and the results confirmed a high ratio between production levels of these cytokines in relapse leprosy compared to all other assessed groups, especially newly diagnosed and untreated MB patients (ratio 59:9; *p<* 0.001; Fig 4F). The concomitant production of IL-1β, IL-6, and TNF, known for inflammatory activity in relapsed patients, without IL-10 production, points to the role of such cytokines in the inflammatory state of the relapsed patients studied. As expected, failure of ML-specific-IFN-γ production was observed in all groups, except in the HC. With respect to all other cytokines evaluated, there were no significant differences between studied groups, except for IL-4 and IL-5, which were found to be increased in newly diagnosed and untreated MB patients (data not shown).

## Discussion

The ethnic origin, gender, and age of the relapsed patients in this study were similar to that of patients showing classical forms of MB leprosy. Our clinical and epidemiological data are comparable to a study carried out in China with a larger number of patients, longer time interval between cure and reemergence of the disease, and greater increase of BI in relapse compared to the FLD [12]. Moreover, among our relapsed patients, the average time for reappearance of disease (12.5 years) corresponded to the findings of other studies [7]. Dapsone monotherapy and 2-year MDT are reported as risk factors for relapse, especially in MB patients [13–15]. In fact, most of our relapsed patients (11/15) received 24-dose MDT. A mean bacillary index (BI) of >3.5 at the onset of disease, observed in our relapsed group, is also consistent with other reports [15,16]. Perhaps immunologically favorable sites such as dermal nerves, lymph nodes, and iris are appropriate places for growth of dormant bacilli responsible for relapse in MB patients, particularly in cases with higher BI. It has been observed that 10% of relapse cases in the lepromatous pole are due to growth of “persister” *M*. *leprae* [17].

Several studies describe a variety of relapse rates [14,18–19], which may be justified by some of the following reasons: difficulties at the diagnosis, time of diagnosis (which should be made as early as possible), and differences in discrimination between RR and relapse. Within this context, none of the relapsed patients studied presented any signs or symptoms that could characterize reactional episodes at the moment of relapse diagnosis. Another important issue concerns the resistance to drugs used at MDT, which may contribute to disease re-emergence. We have previously reported the presence of *M*. *leprae* strains isolated from treated patients with new signs and symptoms of leprosy [20]. Nevertheless, the cases studied in this work did not present drug or multidrug resistance and responded to MDT.

Relapsed patients were less responsive to antigen, thus suggesting a possible role of sustained hyporesponsiveness in triggering the relapse. In these patients, the reduction of CD86 expression in monocytes and DC by ML was significant, while LPS was found to show a similar positive response in all other studied groups, including relapsed patients. The CD80/B7.1 or CD86/B7.2 engagement with the CD28 receptor expressed by T cells has been reported to augment cytokine gene expression, promote cytokine messenger RNA stability, and increase cellular proliferation. Therefore, the absence of costimulation results in hyporesponsiveness or anergy [21]. Previous studies showed that lepromatous leprosy patients presented decreased expression of B7.1, both in monocytes and in cutaneous lesions [22]. Given that B7.1 may utilize a compensatory costimulatory pathway, our previous work has shown that, regardless of the clinical form of leprosy, the addition of anti-CD86, but not anti-CD80, in cultured blood leukocytes led to a down regulation of T cell activation and the proliferative response [23]. In an experimental approach using the Jurkat T-cell leukemic cell line, Dagur and collaborators showed that the ML antigen leads to inhibition of T-cell activation as a result of downstream signaling events, including B7.2/CD28 engagement [24]. Although the addition of ML to PBMC cultures of cured and healthy individuals resulted in a reduction in CD86 expression in both monocytes and DC in our studies, the negative variation of this molecule was higher in the relapsed group, in both cell types. ML-induced negative modulation of CD86 among relapsed patients was approximately two times higher in monocytes than in DC. A similar difference was also observed in other groups, but on a much lower level. Monocytes and DC have distinct and differentially regulated programs for costimulation and the activation and maturation of DC can be directly inhibited by the pathogen itself. Our results suggest that ML may induce tolerance in the relapsed group, acting as a modulating agent and leading to a less competent response, which may interfere with the differentiation of naïve T cells to effector T cells. One possibility for the greater inhibition of CD86 in relapsed patients, compared to newly diagnosed and untreated MB patients, may be the number of skin lesions and BI, which were considerably higher in this our group than the newly diagnosed and untreated MB patients.

The proportion of IFN-γ producing Th1 lymphocytes is low in MB patients, and prior data show that *ex vivo* and *in vitro* responsiveness to ML may be recovered in some MB patients immediately upon treatment [4–6]. In the present study, only in two individuals (>50 pg/mL; 20%) within the cured patients group demonstrated significant levels of IFN-γ in response to the ML antigen (all were MB). Interestingly, these individuals were the most recently discharged patients in the group with 1 and 2-year cures. However, the results from *in vivo* responses were incompatible with those obtained *in vitro*, as no individual demonstrated cutaneous positivity to the LST after treatment. Despite the small number of cured individuals examined in this study at post-MDT follow up, the data suggest that the cure period may influence the *in vitro* immune response, and individuals may appear to be tolerant due to a reemergence of bacillary multiplication over the years. The relapsed patients tested before and after MDT showed the same classical and constant pattern of hyporesponsiveness to lepromatous leprosy, with concomitant failure in IFN-γ production and LST negativity.

Since most of the mature circulating T lymphocytes can be divided into naïve and memory cells, based on their antigenic response [25], one of our goals was to verify the existence of long-term antigen-specific memory T cell responses after discharge from active leprosy and also to characterize the function and magnitude of T cell responses in relapse. Interestingly, we observed an increase in *ex vivo* (UNS) T_CM_ expression (CD62L^+high^) especially in CD4+ cells, but a reduction of CD4+ and CD8+ T_EF_ expression in PBMC from relapsed patients. The same finding was observed in ML-stimulated cultures. As expected, we also observed decreased CD69 expression in ML-specific CD4+ and CD8+ T_N_ and T_EF_ cells in newly diagnosed and untreated MB patients, which was contrary to our observations in some cured individuals and in all PB and HC. Although the group of cured individuals encompassed MB leprosy patients, knowingly with a T cell functional defect, the significant increase in their CD8+ T_EF_ and T_EM_ cells in response to the ML antigen (*p*<0.05) suggests that these subsets may play a role in protection against relapse. T_CM_ cells are antigen-experienced T cells that lack immediate effector function, but can mediate rapid recall responses. These cells are a long-lived memory population that circulate through the secondary lymphatic organs and blood [26]. Although they are important in the formation of protective immunity, our data raise the possibility that, in MB relapsed patients, the accumulation of T_CM_ subsets, which could be due to chronic and persistent antigenic stimulation, may be related to disease progression. It is unclear why similar findings were not observed in newly diagnosed and untreated MB patients. Similar to our findings, Oliveira et al. also demonstrated an increase in CD8+ T_CM_ cells in co-infected HIV/leprosy patients showing an acute inflammatory manifestation referred to as reversal reaction. The authors also suggested that CD8+ T_CM_ cells may contribute to these episodes [27]. Interestingly, the negative CD86 expression on both APC did not prevent the significant increase in both CD4+ and CD8+ T_CM_ cells in our patients in response to the ML antigen. This raises the possibility that, in this group, such cells are less dependent on costimulation for activation but rather on the presence of inflammatory cytokines, especially IL-1b. This cytokine, which has already been shown to inhibit expression of CD86 on ML infected APC [28], may have contributed to decreased effector T cell expansion and may favor leprosy relapse.

The low frequency of CD4+ and CD8+ T_EM_ antigen-specific cells observed in our relapsed group could have resulted from established disease and high bacillary load. In this regard, the increased frequency of CD8+ T_EM_ in the cured group may be an indication of the state of the immune response after treatment. A recent study has shown that individuals cured of cutaneous leishmaniasis presented a significant increase in CD4+ and CD8+ T_EM_ without IFN-γ production in response to the *L*. *brasiliensis* antigen [29].

Although our study did not carry out a direct analysis on the frequency of IFN-γ-producing T cells, this cytokine was not significantly found in the supernatant of PBMC cultures stimulated with ML antigen from relapsed patients, neither before nor after MDT. Specifically, among the relapsed patients studied, the significant increase in T_CM_ induced by the ML antigen was about 10% for CD4+ T cells and approximately 5% for CD8+ T cells. Therefore, the expansion of T_CM_ under persistent antigenic stimulation, as was observed in our relapsed cases, leads us to hypothesize that this population could preferentially experience clonal exhaustion, thus maintaining the chronic infection. Moreover, taking into account that *M*. *leprae* is not a strong inducer of a cellular immune response, probably due to the presence of lipid molecules in the cell walls, our results suggest that the potency of the pathogen can strongly influence the differentiation and fate of T cells, and that the extent of attrition of primed T cells is inversely correlated to the early differentiation of T cells primarily into the central subset. Furthermore, several studies indicate that CD4+ or CD8+ T_CM_ and T_EM_ cells are independent subpopulations that can develop according to the biological environment, primarily the site of cellular activation and the cytokine balance within the environment [30].

In the present study, we also aimed to determine whether IL-10 was able to modulate the differentiation of *M*. *leprae*-specific effector T lymphocytes in relapsed patients. During mycobacterial infections, IL-10 inhibits macrophage function resulting in enhanced intracellular bacterial growth and inhibition of nitric oxide production; indicating its crucial role in mediating chronic infections [31]. In fact, among tuberculosis patients, enhanced levels of serum IL-10, *in vitro* cytokine production, increased expression of Treg cells (CD25+FOXP3+), and inhibition of the Th1 response were recently observed [32]. Surprisingly, there was no or low IL-10 production in our relapsed patients, in contrast to the newly diagnosed and untreated MB patients. For some time, the immune suppression observed in lepromatous leprosy was linked with IL-10 production or by IL-10 gene polymorphisms, conferring susceptibility to leprosy [33,34]. A recent elegant experimental study suggested that the interaction between IL-10 and inducible nitric oxide synthase (NOS) may be responsible for the reactivity of antigen-specific T lymphocytes responses related to leprosy neural damage [35]. In fact, the low levels of IL-10 production in response to ML-stimulated PBMC in our relapsed group may be due to the phase of the infection and the diagnosis, where low levels of this cytokine can modulate the chronic inflammatory state, as observed in the lepromatous leprosy patients. Another recent study showed that, only in a minority of patients, the depletion of IL-10 producing Treg cells was responsible for recovery of the capacity to produce antigen-specific IFN-γ production [36]. This suggests that, in MB leprosy, immunoregulation is more complex and dependent on several factors; however further investigation is required.

In this work, as opposed to the findings related to IFN-γ and IL-10 production, significantly higher levels of the inflammatory cytokines IL-1β, IL-6, and TNF among relapsed patients suggest an inflammatory response consistent with the immunopathogenesis disclosed by this group. Determination of cytokine ratios can be helpful, as their balance may contribute to a possible clinical outcome in chronic infectious diseases. It was previously proposed that a balance between IFN-γ, TNF, and IL-10 could be useful either to the determination of protective immunity or to leprosy pathogenesis [37]. Therefore, the high TNF/IL-10 ratio observed in our relapsed group is probably linked to the pathological response developed by these individuals. Our results show that the pro-inflammatory pattern of majority production of IL-1β, IL-6, and TNF in our relapsed patients remains, although at lower levels, in the cured group. Similar results were also observed in a recent study using the same standard ratio between production of pro-and anti-inflammatory cytokines in patients with mucosal leishmaniasis and individuals post leishmaniasis treatment [38].

In summary, several clinically significant conclusions can be made from our findings. The relapse group studied was previously treated, and some of these patients (about 53%) presented reactional episodes during FLD. The predominance of antigen-specific responses of CD4+ and CD8+ T_CM_ cells in these patients suggests that these cells may modulate the cell migration to skin, while keeping a reduced effector function that is unlikely to kill the pathogen. Additionally, the pattern of TNF and IL-1β production (both *ex vivo* and *in vitro* responses to the ML antigen) may influence clinical presentation in these relapsed patients who demonstrate the highest number of skin lesions and higher bacterial load among the experimental groups studied. Newly diagnosed and untreated MB patients without reactions demonstrate the classic hyporesponsiveness to *M*. *leprae* accompanied by IL-10 production. It is possible that these factors that differentiate the two groups may influence the clinical presentation and immune response observed in the relapsed patients. To date, this is the first work that correlates the profile of immune response with clinical presentation of MB relapsed leprosy patients. The understanding of immunological mechanisms related to relapse could assist in disease control and prevent reactions, since leprosy still poses a significant health and economic burden in developing countries.

## Supporting Information

S1 Table Frequencies of lymphocyte immunophenotypes in PBMC. T_N_: naïve T cells (CD45RO-/CD62L+); T_EF_: effector T cells (CD45RO-/CD62L-); T_CM_: central memory T cells (CD45RO+/CD62L+^high^); T_EM_: effector memory T cells (CD45RO+/CD62L-); UNS: unstimulated cultures; ML: *M*. *leprae*-stimulated cultures; REL: relapsed patients group; MB: untreated multibacillary patients group; CUR: cured patients group; PB: untreated paucibacillary patients group; HC: healthy control group; M: median; IQR: interquartile range (25-75% percentile); PBMC: peripheral blood mononuclear cells. The Kruskal-Wallis test was used for comparison of stimulated cells with unstimulated cells and the Mann–Whitney test was used to group comparisons. *p=* significance level; ^a^ *p* < 0.05 in relation to HC group. ^b^ *p*< 0.05 in relation to MB group.
(DOCX)
