# Correction: Correlation between Central Memory T Cell Expression and Proinflammatory Cytokine Production with Clinical Presentation of Multibacillary Leprosy Relapse

**DOI:** 10.1371/journal.pone.0137445

**Published:** 2015-08-31

**Authors:** Danuza Esquenazi, Iris Maria Peixoto Alvim, Roberta Olmo Pinheiro, Eliane Barbosa de Oliveira, Lilian de Oliveira Moreira, Euzenir Nunes Sarno, Jose Augusto da Costa Nery

## Notice of Republication

This article was republished on Jul 23, 2015, to correct improperly-formatted Greek letters throughout the text. Please download this article again to view the correct version.

## References

[1] Esquenazi D, Alvim IMP, Pinheiro RO, Oliveira EBd, Moreira LdO, Sarno EN, et al (2015) Correlation between Central Memory T Cell Expression and Proinflammatory Cytokine Production with Clinical Presentation of Multibacillary Leprosy Relapse. PLoS ONE 10(5): e0127416 doi: 10.1371/journal.pone.0127416 25992795 10.1371/journal.pone.0127416 PMC4437650

