# Supplementary material for: Correlation between Central Memory T Cell Expression and Proinflammatory Cytokine Production with Clinical Presentation of Multibacillary Leprosy Relapse
Source: PLoS One. 2015 May 19;10(5):e0127416. doi: 10.1371/journal.pone.0127416 (PMC4437650; doi:10.1371/journal.pone.0127416)
Supplement: S1 Table — TN: naïve T cells (CD45RO-/CD62L+); TEF: effector T cells (CD45RO-/CD62L-); TCM: central memory T cells (CD45RO+/CD62L+high); TEM: effector memory T cells (CD45RO+/CD62L-); UNS: unstimulated cultures; ML: M. leprae-stimulated cultures; REL: relapsed patients group; MB: untreated multibacillary patients group; CUR: cured patients group; PB: untreated paucibacillary patients group; HC: healthy control group; M: median; IQR: interquartile range (25-75% percentile); PBMC: peripheral blood mononuclear cells. The Kruskal-Wallis test was used for comparison of stimulated cells with unstimulated cells and the Mann–Whitney test was used to group comparisons. p= significance level; a p < 0.05 in relation to HC group. b p< 0.05 in relation to MB group. (DOCX) [file pone.0127416.s001.docx]

| **Phenotype** | **UNS** | | | | | | | | | | **ML** | | | | | | | | | | |
| --- | --- | --- | --- | --- | --- | --- | --- | --- | --- | --- | --- | --- | --- | --- | --- | --- | --- | --- | --- | --- | --- |
|  | **REL** | | **MB** | | **CUR** | | **PB** | | **HC** | | **REL** | | **MB** | | **CUR** | | **PB** | | | **HC** | |
| **CD3+/CD4+** | M IQR | | M IQR | | M IQR | | M IQR | | M IQR | | M IQR | | M IQR | | M IQR | | M IQR | | | M IQR | |
| T_N_ | 0.3 | 0.1-0.6 | 0.2 | 0-0.5 | 0.4 | 0.06-1.5 | 0.9 | 0.3-1.7 | 0.1 | 0.01-0.6 | 0.9 | 0-1.8 | 0.4 | 0.06-1.9 | 0.95 | 0.5-2.6 | 3.1^b^ | 0.7-4.9 | | 3.0^b^ | 2.1-5 |
| T_EF_ | 1.5 | 0.1-4.4 | 1.0 | 0-6.8 | 1.4 | 0-7.8 | 1.8 | 0.9-11.3 | 3.9 | 0.4-7.5 | 1.7 | 1.1-2.1 | 1.5 | 0.8-3.5 | 4.9 | 3.7-21.1 | 13^b^ | 10-21.2 | | 18.6^b^ | 15.5-22.6 |
| T_CM_ | 6.2ª,^b^ | 0.2-27.9 | 0.2 | 0-1.7 | 1.1 | 0.1-2.1 | 0.4 | 0-2.1 | 0.5 | 0.04-1.0 | 16.7ª,^b^ | 6.5-24 | 0.6 | 0.07-1.8 | 2.3 | 0.3-4.1 | 1.6 | 0.3-3.0 | | 1.4 | 0.3-2.6 |
| T_EM_ | 0.8 | 0-2.4 | 1.0 | 0.06-2.0 | 0.4 | 0-1.8 | 1.7 | 0.8-3.5 | 0.6 | 0-3.0 | 1.6 | 0.9-2 | 1.2 | 0.8-2.7 | 1.3 | 0.4-3.4 | 9.9^b^ | 4.4-15.7 | | 15.3^b^ | 7.1-19.1 |
| **CD3+/CD8+** |  | | | | | | | | | | | | | | | | | | | | |
| T_N_ | 1.3 | 0.6-1.8 | 0.4 | 0.05-1.1 | 1.9 | 1.3-3.7 | 2.3 | 0.5-3.4 | 1.2 | 0.3-2.4 | 3.2 | 2-4.7 | 2.2 | 1.4-3.9 | 8.4^a^ | 1-14.3 | 25.8 | | 6.0-41.1 | 27.5^b^ | 9-56.9 |
| T_EF_ | 1.1 | 0.2-4.1 | 0.5 | 0-2.1 | 2.1 | 1.3-8.7 | 3.2 | 2.4-19.0 | 5.5 | 0.3-12.7 | 1.6 | 0.9-6.4 | 1.6 | 1.2-3.2 | 13.6^a^ | 0.9-20.6 | 22.8 | | 9.0-36.1 | 25.5^b^ | 3.1-32.3 |
| T_CM_ | 9.6ª,^b^ | 0.4-27.3 | 1.2 | 0-1.6 | 1.3 | 0.8-2.3 | 1.1 | 0-2.2 | 0.7 | 0-1.0 | 14.5ª,^b^ | 6-27.5 | 1.0 | 0-2.8 | 1.7 | 0.8-2.7 | 1.09 | | 0-3.2 | 1.2 | 0-2.5 |
| T_EM_ | 1.8 | 0.6-4.6 | 0.6 | 0-3.4 | 2.5 | 1.2-3.7 | 4.2 | 0.4-14.2 | 4.6 | 2.4-8.5 | 2.2 | 1.2-10.4 | 2.4 | 0.7-5.8 | 8.3^a^ | 0.4-19.6 | 12.7^a^ | | 6.8-27.1 | 31.3^b^ | 23-48.9 |
|  |  |  |  |  |  |  |  |  |  |  |  |  |  |  |  |  |  | |  |  |  |
